# The Characteristic and Changes of the Event-Related Potentials (ERP) and Brain Topographic Maps before and after Treatment with rTMS in Subjective Tinnitus Patients

**DOI:** 10.1371/journal.pone.0070831

**Published:** 2013-08-12

**Authors:** Haidi Yang, Hao Xiong, Rongjun Yu, Changming Wang, Yiqing Zheng, Xueyuan Zhang

**Affiliations:** 1 Department of Otolaryngology, Sun Yat-sen Memorial Hospital, and Institute of Hearing and Speech-Language Science, Sun Yat-sen University, Guangzhou, China; 2 School of Psychology and Center for Studies of Psychological Application, South China Normal University, Guangzhou, China; 3 National Key Laboratory of Cognitive Neuroscience and Learning, Beijing Normal University College of Information Science and Technology, Beijing, China; University G. D'Annunzio, Italy

## Abstract

**Objectives:**

To compare the event-related potentials (ERPs) and brain topographic maps characteristic and change in normal controls and subjective tinnitus patients before and after repetitive transcranial magnetic stimulation (rTMS) treatment.

**Methods and Participants:**

The ERPs and brain topographic maps elicited by target stimulus were compared before and after 1-week treatment with rTMS in 20 subjective tinnitus patients and 16 healthy controls.

**Results:**

Before rTMS, target stimulus elicited a larger N1 component than the standard stimuli (repeating sounds)in control group but not in tinnitus patients. Instead, the tinnitus group pre-treatment exhibited larger amplitude of N1 in response to standard stimuli than to deviant stimuli. Furthermore tinnitus patients had smaller mismatch negativity (MMN) and late discriminative negativity (LDN)component at Fz compared with the control group. After rTMS treatment, tinnitus patients showed increased N1 response to deviant stimuli and larger MMN and LDN compared with pre-treatment. The topographic maps for the tinnitus group before rTMS -treatment demonstrated global asymmetry between the left and right cerebral hemispheres with more negative activities in left side and more positive activities in right side. In contrast, the brain topographic maps for patients after rTMS-treatment and controls seem roughly symmetrical. The ERP amplitudes and brain topographic maps in post-treatment patient group showed no significant difference with those in controls.

**Conclusions:**

The characterical changes in ERP and brain topographic maps in tinnitus patients maybe related with the electrophysiological mechanism of tinnitus induction and development. It can be used as an objective biomarker for the evaluation of auditory central in subjective tinnitus patients. These findings support the notion that rTMS treatment in tinnitus patients may exert a beneficial effect.

## Introduction

Subjective tinnitus is defined as a sensation of noise-like ringing or roaring that is purely subjective phantom phenomenon without any sound source. It has been estimated that approximately 10–15% of the population are affected by chronic tinnitus [1]. The number may be increasing in industrialized and aged countries. About 1 to 3% of the tinnitus population is quite distressing because it causes severe impairments in the quality of life and therewith related high socioeconomic costs (e.g., missing working hours or early retirement). Some tinnitus patients get accustomed to it. But others develop several psychiatric symptoms such as depression and insomnia problems with auditory perception [3] or poor general and mental health [2]–[3]. Unfortunately at the current stage there is no effective therapy that can reliably eliminate the tinnitus sensation. The principal pathophysiological mechanism causing chronic distressing tinnitus is still poorly understood and unclear.

Tinnitus is linked to abnormal changes at one or more levels along the auditory pathway. Human brain imaging studies have identified altered tinnitus-related activity in auditory areas, including the inferior colliculus and auditory cortex [4]–[6]. The central auditory system appears to increase its gain to compensate for the reduced sensorineural input from the cochlea induced by acoustic trauma, ototoxic agents or other causes. Hyperactivity can manifest itself as a phantom sound of tinnitus, as well as hyperacusis or intolerance of loud sounds. Apart from hyperactivity, tinnitus-related changes in the auditory system also include increased neural synchrony and bursting activity. Similar bursting activity has been reported for the inferior colliculus after salicylate treatment [7] or cisplatin treatment [8]. Animal models have corroborated this explanation, pointing to the presence of reorganized tonotopic maps and increased spontaneous neural activity or synchrony in the auditory cortex at the origin of tinnitus [9]. There is also an increasing amount of evidences from both animal and human studies supporting that tinnitus is related to enhanced excitability of auditory cortical regions [10], putatively leading to spurious spontaneous synchronization [11]. The direct measurement of neuronal spiking in humans is possible only by the use of invasive methods. Alternatively, functional changes in auditory cortex activity can be revealed in tinnitus patients using functional magnetic resonance imaging (fMRI) or high-resolution electroencephalography (EEG) [12]. fMRI and positron emission tomography (PET) have revealed differences in sound-evoked responses between tinnitus and non-tinnitus groups in the cortex [13]. The ERPs can reflect the functional state of the auditory cortex as well, but the results were not completely unified. Jacobson and McCaslin [14] observed significantly smaller N1 amplitudes in tinnitus patients as compared to subjects without tinnitus, but the results could not explain hyperactivity and increased neural synchrony or bursting activities in the auditory system. Norena et al. [15] had found that N1 latency was shortened in patients with bilateral tinnitus and N1-P2 complex amplitude was increased for the tinnitus ear than the non-tinnitus ear in patients with unilateral tinnitus. Actually, tinnitus was also considered as a sentience epilepsy or auditory hallucinations, and thus may have similar mechanisms and ERP characteristics with other mental illnesses. Gene-Cos et al. [16] reported that mismatch negativity (MMN) amplitudes tended to be larger in patients with epilepsy than in healthy controls. One could hypothesize that the higher amplitudes observed in epileptic patients indicate increased activation of the same neuronal population as in controls, or that extra neuronal circuits are activated in epileptic patients [17]. Taken together, the existing literature provides discrepant accounts of how MMN is affected in epilepsy patients. Areas involved in auditory processing are shown to be abnormal in schizophrenia. Structural and metabolic abnormalities of the auditory cortices are observed. For example, Dierks et al. [18] found an increased blood oxygen level-dependent (BOLD) signal in Heschl's gyrus during the hallucinations of schizophrenia patients, suggesting the involvement of primary auditory areas in auditory verbal hallucinations. These previous studies provided evidences to exploit the mechanisms and clinical intervention of tinnitus.

Transcranial magnetic stimulation (rTMS), a non-invasive technique for depolarizing cortical neurons based on the principle of electromagnetic induction (Barker et al. 1985), has recently gained popularity not only as a research tool but also as a possible treatment for chronic tinnitus [19]–[21]. Khedr found 1 and 25 Hz rTMS contralateral to the side of tinnitus have a greater beneficial effect on symptoms than either ipsilateral stimulation by their study of Contralateral versus ipsilateral rTMS of temporoparietal cortex for the treatment of chronic unilateral tinnitus [22]. Based on the finding that 1 Hz rTMS in general reduces cortical excitability, 1 Hz rTMS has been extensively used over the temporo- or temporoparietal cortex as a treatment tool for chronic tinnitus during recent years. But it is still unclear that how this technique could alleviate tinnitus symptoms and how to further improve its efficacy.

In a recent treatment study Okamoto and colleagues [23] demonstrated a reduction of the perceived tinnitus loudness after individualized auditory stimulation accompanied by reduced auditory cortex activity amplitudes. Isabel Lorenz [24] also indicated the enhanced auditory cortical activity (reflected in the ASSR and the N1) is reduced after rTMS. Unfortunately, the influences of rTMS on other components evoked by auditory stimuli besides ASSR and N1 in tinnitus patients have not yet been investigated. Few studies have yet investigated the neurophysiological differences in ERP characteristics before and after treatment with rTMS between tinnitus patients, neither, although this could lead to a better understanding of pathological auditory neural activity. Thus the primary goal of the present study was to reveal the impacts of rTMS on auditory cortical activities and compare the features of ERP components and brain topographic maps before and after rTMS treatment on tinnitus patients. These ERP characteristics could help explore the tinnitus electrophysiological mechanisms and evaluate treatment effects and make direct inferences about the neural basis revealed by ERP of behavioural phenomenon caused by rTMS.

## Methods

### 2.1. Ethics Statement

The present study protocol was approved by Institutional Review Board on experimental ethics committee at Sun Yat-sen University. All subjects provided written informed consent to participate.

### 2.2. Subjects

20 tinnitus patients (10 males, 10 females,ranging from 31 to 60 yr)and sixteen healthy subjects without tinnitus (10 females and 6 male,ranging from 20 to 45 yr) took part in the experiment. All the subjects were right-handed with normal or corrected-to-normal vision. All of the control group subjects were free of DSM-IV Axis I diagnoses of disorders. None of them had neurological diseases, a history of any substance dependence, or a history of clinically significant head trauma. Audiometry and otoscopy were performed at enrolment. We applied pure tone audiometry testing to quantify the average of pure-tone hearing threshold (the average of 0.5,1,2,4 KHz), a.k.a pure tone average (PTA). Normal hearing is defined as PTA lower than 20 dB. Mild hearing loss is defined as PTA between 21 and 40 dB. All subjects in the control group have normal hearing. In the tinnitus group, 12 out of 20 have mild hearing loss (see Table 1). Since our experiment involves auditory stimuli, we only selected patients with normal hearing or mild hearing loss and exclude patients with severe hearing loss. Tinnitus handicap inventory (THI) was used to grade tinnitus severity [25], [26]. The THI is a self-report measure that can be used in a busy clinical practice to quantify the impact of tinnitus on daily living. We used the 25-item beta version of the THI with the items grouped into functional, emotional, and catastrophic subscales. It has been shown that the total scale yielded excellent internal consistency reliability (Cronbach's alpha  = .93) [26]. The average THI score for tinnitus patients was 68±11 (mean ± SE).

**Table 1:** Demographic information for the patient sample and control sample.

Measure	TP (n = 20)		HC (n = 16)		statistics
	Mean	SD	Mean	SD	P
Age (year)	43.2	13.1	42.5	12.7	0.45
Gender (male)	10	n.a.	6	n.a.	0.72
Illness duration (year)	3.2	1.5	n.a.	n.a.	n.a.
THI	68.3	11.6	n.a.	n.a.	n.a.
Mild hearning loss	12	n.a	0	n.a.	n.a.
Tinnitus frequency (Hz)	4415	2531	n.a.	n.a.	n.a.
Tinnitus in right ear	5	n.a	n.a.	n.a.	n.a.
Tinnitus in left ear	8	n.a	n.a.	n.a.	n.a.
Tinnitus in bilateral ear	7	n.a	n.a.	n.a.	n.a.

Note Mean and standard deviation are provided for continuous variables (e.g., age, illness duration, tinnitus frequency and THI). TP  =  tinnitus patients. HC  =  healthy controls. THI  =  Tinnitus Handicap Inventory Score.

### 2.3. rTMS procedures

Individual resting excitability thresholds for left motor cortex stimulation were determined each day, according to International Guidelines, prior to the rTMS session, by using the same coil and stimulator. Active stimulation was administered using a standard Mag-Stim Super Rapid stimulator system and figure-eight coil positioned midway between the left temporal (T3) and left parietal (P3) EEG electrode sites. The contralateral temporoparietal region was targeted, in accordance with other studies. Repetitive rTMS consisted of 2000 stimulations/day (20 trains of 100 stimuli with an intertrain interval of 1 minute) at 1 Hz and 110% of the motor threshold (MEP), for five consecutive days (from Monday to Friday) per week and lasted 2 weeks. A high number of stimuli/day was applied because of the previously suggested dose dependency of tinnitus alleviation by rTMS.

### 2.4. EEG Recording and processing procedure

During the experiment, they were sitting in a quiet dark room with arms relaxed and were required to watching “silent” films. Electroencephalography (EEG) data for the sixteen normal participants and twenty patients was collected from an EGI system (Electrical Geodesics Inc.). For the patients, the EEG data before and after rTMS treatment was both recorded. There were 128 channels on EGI's HydroCel Geodesic Sensor Net and Cz was used as the reference channel. All the electrode impedances were detected and maintained at less than 5 kΩ before test. The odd-ball stimuli pattern with pure tone (1500 Hz-1000 Hz, 75 dB, 50-msec duration with a shaped 5-msec rise and fall time) was used in this study (via Eprime 2.0, Psychology Software Tools, Inc.). A regular stimulus 1000 Hz was presented at 85% of the trials, together with a target stimulus 1500 Hz at a 15% of the trials. The whole task consisted of a total of 1000 auditory stimuli with random inter-stimulus intervals (ISI) ranging from 850 ms to 1450 ms. Stimuli were delivered through two loudspeakers at a distance of 100 cm from the subjects, with around 75 dB at both ears. The two speakers were positioned so that they are angled 45 degrees to the ears and 90 degrees to each other. We avoided using headphones or insert phones since they may induce temporary hearing loss. Subjects sat in a comfortable chair in a sound-attenuated room, were watching silent films and were not asked to make explicit decisions about the category information of the stimulus or to respond to any auditory stimulus during test. At the end of each session, all subjects reported that they were awake during the whole experiment and paid attention to the film.

The EEG data was continuously recorded with the sampling rate of 250 Hz. Then, the EEG data was bandpass filtered using a casual finite impulse response (FIR) filter (0.15∼40 Hz, linear-phase shift). To get rid of the eye-blink noise, independent components that were sensitive to eye blinks were identified and removed from the EEG data with the Independent Component Analysis (ICA) approach. The EEG data was then baseline-corrected and epoched by stimulus conditions. The filtering, ICA, baseline-correction and epoch functions were all supported by the modules in EEGLAB, a Matlab-based toolbox. Those trials contaminated by head movement noises were visually inspected and rejected. All responses at individual electrodes were referred to the montage reference value. Finally, the trials from each stimulus category of each subject were grandly averaged and several typical event-related potential components, including P1, N1 and P2, evoked by auditory stimuli were obtained. The difference waveform between deviant and standard stimuli was also calculated to determine the mismatch negativity (MMN) and LDN. Both MMN and LDN reflect processing of stimulus change. The MMN indicates an automatic neural-mismatch process triggered by the sensory input from a rare deviant stimulus in the presence of a neural trace of the frequent standard stimulus. The LDN is a late negativity peaking at about 600–800 ms and indicates nonautomatic or controlled processes that are probably activated through task manipulation or counting of deviant stimuli.

P1, N1, P2, MMN and LDN are defined by their polarities and latencies. The P1 component to target and regular events was identified as the positive range between 50 and 100 msec, N1 was between 80 and 120 msec, P2 was between 150 and 300 msec. Different from P1 and N1, MMN was a difference waveform between deviant and standard stimuli, which was identified as a negative component between 100 and 250 msec after subtracting the deviant waveform by standard waveform. Likewise, another difference waveform LDN between deviant and standard stimuli was selected between 400 and 600 msec. The peak values of ERP components were identified by computer algorithms by seeking the most positive or negative peak within a time window. The ERP amplitudes within the corresponding windows were also averaged and used for brain topographic maps.

## Results

The characteristics of the patients and controls who completed the experiment are summarized in table 1. The majority of patients did not complain of side effects from rTMS, apart from a slight transient headache on the stimulation site which did not require pharmacological treatment.

In this study, we focused on several typical ERP components and compared their differences between normal subjects and patients with tinnitus. We also compared tinnitus patients before and after rTMS treatments. In tinnitus patients, more negative N1 components for standard stimuli and lower MMN amplitudes were observed, and the latency of MMN at Fz was about 20 msec delayed in tinnitus patient group before rTMS-treatment compared with that in the control group (but no significantly different). The amplitude differences in N1 by standard stimuli, MMN and LDN are all statistically significant (p<0.05). The N1 for target stimuli did not significantly differ between patients and normal controls (see Table 2).

**Table 2:** The peak latencies and amplitudes of N1, P2, MMN and LDN at Fz between tinnitus before or after rTMS and control group.

	Number	N1		P2		MMN		LDN
		Latency	Amplitude	Latency	Amplitude	Latency	Amplitude	Amplitude
CG	16	107±6	−1.4±1.0	202±30	1.3±0.8	163±18	−1.6±0.7	−1.2±0.5
TG before rTMS	20	110±8	−2.0±1*	211±24	1.6±1	182±26	−0.5±0.3^**^	−0.3±0.1^**^
TG after rTMS	20	109±5	−1.5±1.2	205±20	1.5±0.6	174±20	−1.3±0.5	−1.0±0.4

* Correspond to P<0.05: significant difference: while** to P<0.01;NS: no statistical difference; CG: control group; TG: tinnitus group.

**Figure 1:**
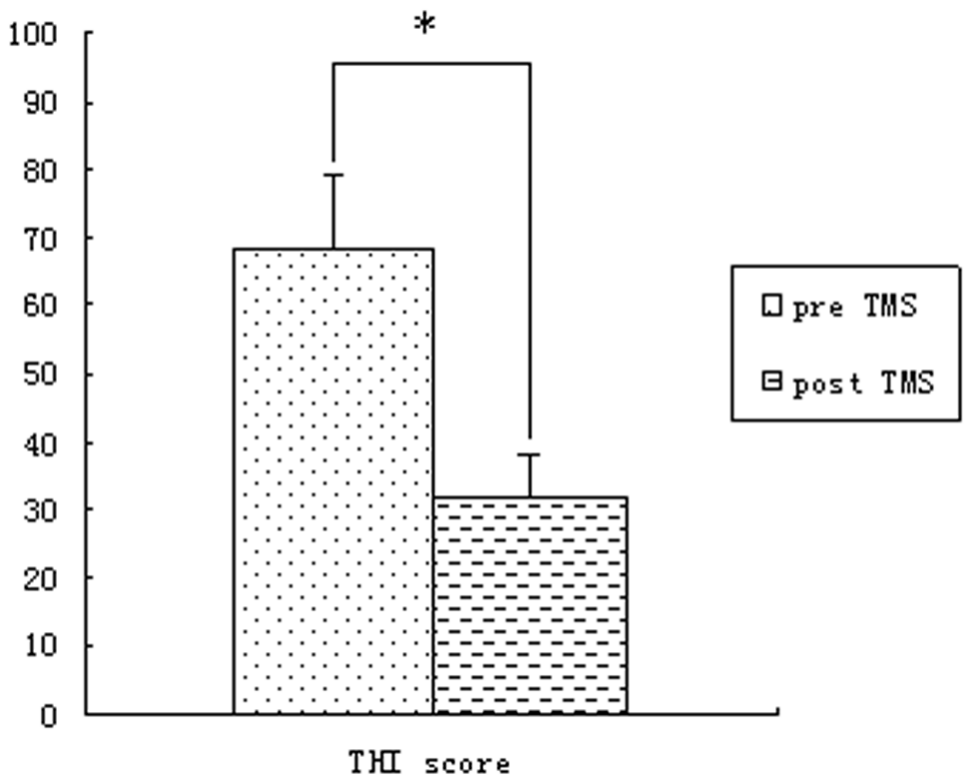
The THI score in post-treatment as compared to themselves before treatment (from THI 68±10 pre-treatment decrease to 32±6 post-treatment, P<0.01).

**Figure 2:**
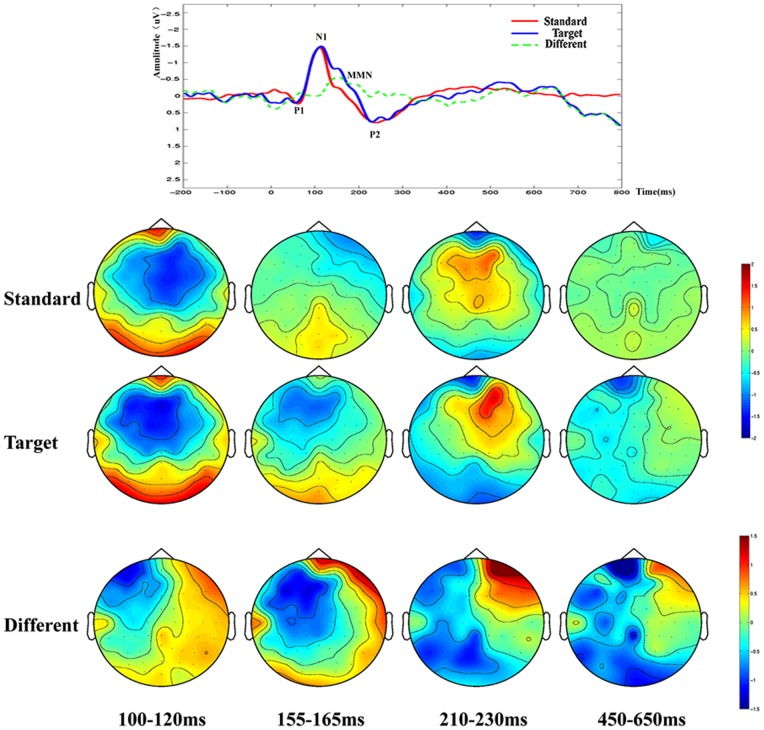
The ERP waveforms and brain topographic maps of tinnitus patients before rTMS. N1, P2 and MMN are denoted here, but no obvious LDN is present in the ERP results of this group.

**Figure 3:**
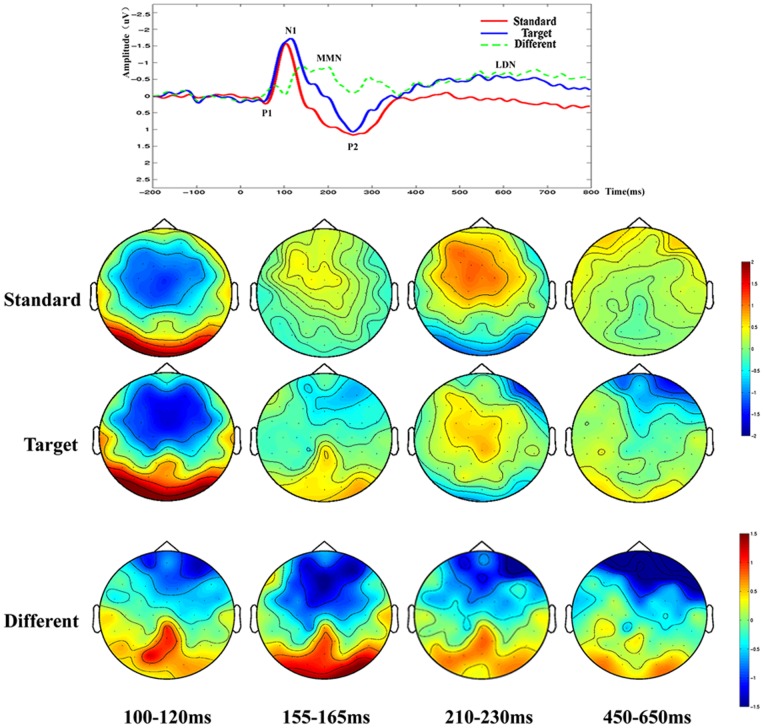
The ERP waveforms and brain topographic maps oftinnitus patients after rTMS. N1, P2, MMN and LDN are denoted in this figure, and the amplitudes and latencies of N1 and MMN are different from those by normal controls and tinnitus patients before rTMS.

**Figure 4:**
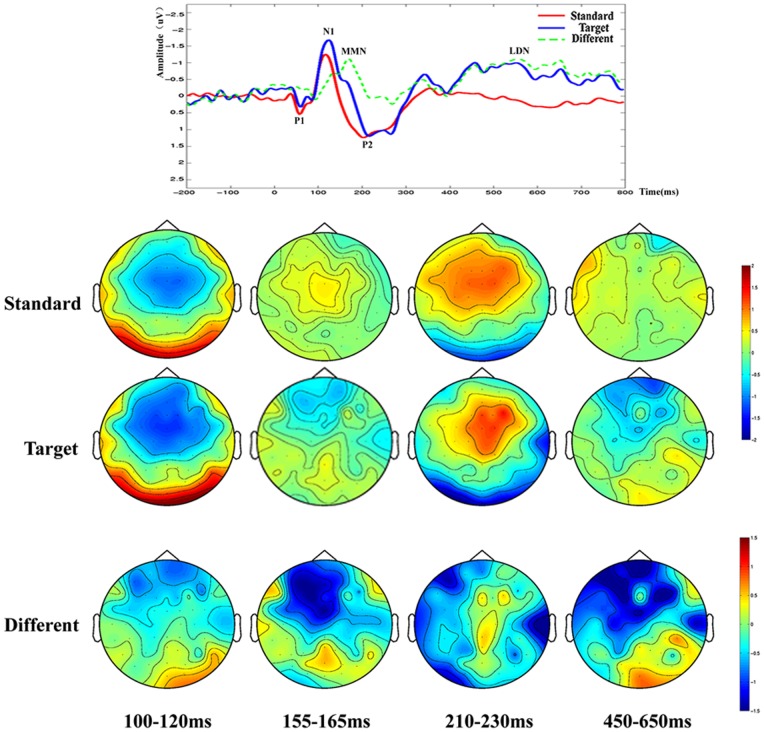
The ERP waveforms and brain topographic maps of normal control group. N1, P2 and two contrast waveforms between stimuli conditions MMN and LDN are denoted in this figure along with their brain topographic maps below.

Similar differences were observed between the ERPs acquired before and after rTMS -treatment in the patient group. As shown in THI scores, the patients exhibited significant improvement in post-treatment phase as compared with before treatment (from THI 68±10 pre-treatment decrease to 32±6 post-treatment, t = 4.56, P<0.01, see Fig 1). This was reflected as a significant higher P1 peak amplitudes and higher MMN in post-treatment tinnitus patients. LDN was another ERP component that may contribute to distinguishing tinnitus patients from normal subjects. LDN was so small that it did not appear in the contrast waveforms of tinnitus patients beforer TMS-treatment (see Fig. 2), However after TMS, LDN showed a significant high amplitude in tinnitus patients after rTMS treatment (see Fig. 3) and in the normal control group (see Fig. 4) (p<0.01). The amplitude and latency features in ERP components may serve as an index reflecting whether the tinnitus effects have been alleviated to the states of normal subjects.

In addition to the ERP amplitudes differences, the spatial distribution features of ERP peaks are also information about tinnitus. The topographic maps in Figure 2 for the tinnitus group before rTMS -treatment demonstrated global asymmetry between the left and right cerebral hemispheres with more negative ERP amplitudes in left side and more positive ERP amplitudes in right side. In contrast, the brain topographic maps for patients after rTMS -treatment and normal subjects seem roughly symmetrical. It is also observable in figure 3 that brain topographic maps of MMN at post-treatment phase were closer to those by normal controls. For example, according to the spatial distribution features of MMN, the frontal cortex were found more negative and the posterior cortex were more positive on both normal subjects and post-treatment patients. The morphological characteristics of the spatial distribution of ERP features may suggest that the neural mechanisms of tinnitus patients after rTMS were recovered to the degree of approaching those of normal hearing subjects (Fig 2,3,4).

## Discussion

The ERP components we used in this study may reflect several stages of auditory perception processes, which are generated from auditory thalamocortical and corticocortical pathways, including the primary and association cortices [27]–[28].

In our results, we found patients with tinnitus exhibited specific patterns of cortical activities that differed from control subjects in several ways. First, the amplitude of N1 by regular stimuli was enhanced in tinnitus patients, resulting in there were not obvious differences in N1 peak amplitudes elicited by regular stimulus and target stimulus. Actually, in the normal group, target stimulus presented within a repetitive sequence often elicit larger N1 components than the repeating sounds [29]. This N1-effect is believed to be attributed to the refractoriness, habituation, or adaptation of neural populations that is more sensitive to the irregular properties of sounds [30]. The larger N1 in tinnitus patients may be caused by the increased neural synchrony due to the reorganization of cortical tonotopic maps of neurons, resulting in higher baseline activities in tinnitus patients and making it impossible to increase the neural activities to process novel stimuli (deviant stimuli) any longer. With respect to the adaptation hypothesis, such findings may reﬂect poor neuronal adaptation in the temporal lobe such that repeated presentation of standard stimuli does not lead to reduced responses.

Second, the tinnitus patients had smaller MMN amplitudes at Fz compared with the control group and themselves after clinical treatment with rTMS. MMN is thought to reflect a sensory memory-based deviance-detection process (for a recent summary on the interpretation of the ERP component). Two major competing hypotheses, the model adjustment hypothesis and the adaptation hypothesis, are considered below in relation to the current findings in this study. To date, the most commonly suggested mechanism underlying MMN generation is a pre-attentive sensory memory mechanism posited to automatically compare present auditory input and memory traces of previous sounds. More specifically, it has been suggested that MMN may reﬂect on-line modifications of a perceptual model that is updated when auditory input does not match its predictions, a hypothesis known as the model-adjustment hypothesis. Deviants may also capture attention, that is, leading to an involuntary attention-shift towards the deviating stimulus. Miho Miyajima et al had observed in temporal lobe epilepsy(TLE) patients the MMN component had smaller amplitude at frontocentral sites than in controls. Gene-Cos et al [16] agreed that prolonged MMN duration might point to the difficulty mainly in “the closure mechanism of the MMN process”, which indicated that this information processing dysfunction related to concentration and memory difficulties. Tinnitus can been considered as a sensitive epilepsy and audiology hallucination, and thus reduced MMN at frontocentral sites may be interpreted in terms of increased activation of the same neuronal population as in controls, or activation of extra neuronal circuits. These patients may spend more time evaluating stimulus novelty than controls and may experience difficulty switching attention from one stimulus to another by noise tinnitus sound. That is, a larger number of synchronously activated frontal neurons may be required for successful automatic attention-switching in tinnitus patients than in controls, due to the impairment of an initial sensory memory mechanism in the temporal lobe. An alternative mechanism recently proposed by Jääskeläinen et al. [31] suggests that MMN results from a much simple mechanism of local neuronal adaptation in the auditory cortex. But after treatment with rTMS, the MMN amplitude can be increased to approach to those of the control group, which demonstrate this function is getting well following the improvement of tinnitus revealed by THI (68±10 for pre-treatment rTMS vs 32±6 for post-treatment rTMS).

Third, late ERP components representing normal function of emotion in tinnitus patients are also different from those in control groups. Compared to standard stimuli, deviant stimuli may also elicit a late negative component, for which we will use the descriptive term late difference negativity in the following (LDN) [32]. In our control group, obvious LDN could be elicited. This component usually peaks around 400–500 ms following deviance onset. However, it is absent in tinnitus patients and gets recovered when those patients were treated by rTMS for 1 week. The LDN might reflect certain aspects of sound discrimination, since it is elicited in an oddball paradigm in response to deviant sounds. It might be associated with “sensitisation processes” after a stimulus change and may serve as an automatic preparatory process for the detection of any additional changes. However, the functional role of LDN in information processing is far from clear. The absent of LDN in tinnitus patients may be interpreted by the damaged higher-order sound change processing function [33], or the attentional reorientation to the task with being distracted of tinnitus sound [34], [35] or abnormality in the so-called sensitization negativity which would reflect an automatic preparation for the detection of subsequent stimulus changes. The fact that LDN can be recovered after rTMS -treatment indicated the plasticity of this function could be modulated by rTMS.

In addition to the latencies and amplitudes of ERP components, the spatial distribution represented by the brain topographic maps could also reveal the abnormal asymmetrical characteristics and thus the abnormal mechanisms of tinnitus patients. The morphological characteristics of the waveform by tinnitus patients after rTMS was closer to morphology by normal hearing subjects, which indicates that the possible reorganization and plasticity of central auditory cortex may exist in patients with tinnitus and the changes in tinnitus may be correlated with the subjective perception. The results were compatible with the fMRI findings of De Ridder [36] and PET imaging studies by Plewnia C [37] showingthat fMRI activation was greatest in the auditory cortex ipsilateral to the side of symptoms when patients with unilateral tinnitus listened to music because of greater baseline activities in the contralateral auditory cortex of tinnitus patients, leaving it less capable of increasing activation during sound stimulation (i.e. a saturation model).

Some limitations in our study are worth mentioning. First, it is possible that the behavioral and physiological results we found may be caused by the placebo effect of rTMS since we did not apply rTMS on healthy controls. However, behaviorally, healthy controls have intact hearing ability and there is no room for improvement. Nevertheless, future studies may further examine whether rTMS influence the neurobiological responses in healthy subjects using the current paradigm. Second, although our patients do not have psychiatric disorders at the clinical level, they may experience high degree of depression and other mental health problems than controls. It is well known that patients with tinnitus have less quality of mental health than controls [2]–[3], which might be a confounding factor. More detailed quantitative measure of psychiatric symptoms should be carried out in future studies. Finally, our paradigm did not capture other ERP components such as P300. The P300 has been shown to be elicited by deviant stimuli in the classic oddball task. We used a passive task in which participants were engaged in the movies rather than the sound. Future studies may ask participants to pay close attention to the experimental conditions and examine the P300 effects.

As described above, behavioral performances as well as the N1, MMN and LDN of ERP component showed significant improvement as a result of rTMS modulations over auditory cortex. After rTMS, the amplitude of N1, MMN and LDN, along with THI, was decreased in tinnitus group. Both relevant ERP components amplitudes and brain topographic maps were closer to those exhibited by normal controls. It suggested there may be correspondence between tinnitus behavioural performance (revealed by THI), pre-attentive processing (revealed by MMN) and negative emotions (revealed by LDN) in tinnitus patients. In a word, the ERP characteristics may help distinguish tinnitus patients from normal subjects and those ERP from patients receiving rTMS treatment seem closer to those from normal subjects. The amplitude of MMN and LDN may become a useful objective index to evaluate the prognosis and treatment effect of tinnitus.
